# Prescribing Practices, Motivations, and Behaviors Contributing to Prescription Stimulant Misuse Among Medical Students at an Allopathic Medical School: Cross-Sectional Survey Study

**DOI:** 10.7759/cureus.104712

**Published:** 2026-03-05

**Authors:** Hannah B Short, Erik Lehman, Sruthi Nelluri, James Latronica, Bhavna Bali

**Affiliations:** 1 Radiology, Penn State College of Medicine, Hershey, USA; 2 Public Health Sciences, Penn State Health Milton S. Hershey Medical Center, Hershey, USA; 3 Addiction Medicine, Penn State Health Milton S. Hershey Medical Center, Hershey, USA; 4 Psychiatry, University of Pittsburgh Medical Center, Pittsburgh, USA

**Keywords:** adderall misuse, attention deficit hyperactivity disorder (adhd), drug diversion, malingering, medical students, medication diversion, prescribing practices, prescription stimulants

## Abstract

Objective

The rate of prescription stimulant misuse has steadily risen over the past few decades. Medical students are at significant risk; however, limited data exist surrounding misuse in this population. This cross-sectional survey aimed to investigate prescribing practices, behaviors, and motivations contributing to medical student prescription stimulant misuse.

Methods

An electronic survey was distributed via university email to all medical students enrolled at an allopathic medical school. Survey assessed prescribing practices, malingering, diversion, provider counseling, perceived medication safety, knowledge of adverse effects and consequences of diversion, pressure to use medication, and normality of peer misuse. Sample t-tests, chi-square tests, and Kendall’s Tau-b tests were used for analysis.

Results

Of the approximately 600 students eligible for inclusion in the study, 290 (48.3%) completed the survey. 256 (88.3%) respondents reported no prescription stimulant use, 29 (10.0%) reported use with a prescription, and 5 (1.7%) reported use without a prescription. 12 (41.4%) of the 29 respondents with a prescription received their first prescription while in medical school, 3 (10.3%) were prescribed without a complete evaluation for attention deficit hyperactivity disorder (ADHD), and 2 (7.4%) engaged in malingering. A total of 16 users (55.2%) received medication counseling, and 15 users (51.7%) received legal counseling at the time of prescription. Receiving legal and medication counseling was associated with a significantly higher average understanding of consequences of diversion (p = 0.012) and medication side effects (p = 0.017), respectively. There were no significant differences in motivations for use or behaviors of use between users with and without a prescription. The year of medical school was significantly associated with stimulant use behavior (p = 0.03). Students experiencing greater pressure to use medication to compete academically were significantly more likely to indicate an interest in matching into psychiatry (p = 0.036), obstetrics & gynecology (p = 0.025), interventional radiology (p = 0.044), urology (p < .0001), and physical medicine & rehabilitation (p = 0.050) residencies. Among non-users, there was a statistically significant positive correlation between pressure to use medication and both the frequency of witnessing peers use medication without a prescription (+0.336, p < 0.0001) and the likelihood of malingering (+0.422, p < 0.0001).

Conclusions

The prevalence of non-prescription stimulant use was lower than what has been reported in previous studies; however, cases of malingering and potentially inappropriate prescribing of stimulants were observed among students with prescriptions. Efforts should be taken to increase provider counseling on the medical risks of use and the implications of diversion. Mitigating the perceived normalcy of stimulant misuse may be an effective strategy for reducing misuse among medical students.

## Introduction

Prescription stimulants, such as amphetamine and methylphenidate, are prescribed to treat conditions such as attention deficit hyperactivity disorder (ADHD) and narcolepsy [1]. Amphetamines improve alertness and attention by increasing the concentration of norepinephrine in the prefrontal cortex and dopamine in the striatum [1]. Compared to placebo, prescription stimulants demonstrate considerable efficacy in the treatment of ADHD [2]. Their use also reduces the risk for motor vehicle crashes, substance use disorders, criminal acts, and unintentional physical injuries [3,4]. Short-term adverse effects include irritability, decreased appetite, stomach upset, elevated blood pressure, and insomnia. There is conflicting data on whether prescription stimulants are associated with an increased risk of cardiovascular disease. Some studies have found there to be an increased risk of transient ischemic attack (TIA), ventricular arrhythmias, and hypertension among adults; however, other studies have found no association [3,5]. When considering the efficacy, tolerability, and safety of prescription stimulants and non-stimulants, prescription stimulants are still considered first-line treatment for ADHD [2].

Prescription stimulants are classified as Schedule II drugs, meaning they provide therapeutic medicinal benefit but also pose a significant risk for misuse [6]. The prevalence of prescription stimulant misuse, particularly among college students and young adults, has steadily risen over the past decades [7]. This trend exists in the setting of a significant overall increase in prescribing rate [8]. For instance, the annual prevalence of non-medical use of stimulant medication among U.S. college students increased from 6.6% to 9.3% between 2000 and 2011 [7]. Adults 19-28 years of age outside of college displayed a similar trend, with rates of misuse increasing from 5.4% to 7.2% [7]. Rates have remained comparably high in more recent studies. In 2020, a national survey investigating adolescent and adult drug use reported a 6.5% prevalence of prescription stimulant misuse among college students [9]. Although an upward trend in misuse has been observed in other age groups, studies have consistently found the risk to be greatest among adults 18 to 25 years of age [10].

Experiencing euphoria, achieving prolonged wakefulness, and promoting weight loss are frequently cited motivations for misuse [11]. The most common incentives, however, are improved academic performance and enhanced cognitive functioning while studying [12,13]. Medical students are at an even greater risk for stimulant misuse given their high academic workload and pressure. In fact, most medical students who report using stimulants state they began their first year of medical school to improve alertness for competitive exams [14]. The rate of prescription stimulant misuse among medical students is far greater than that found in the general adult population. For instance, a 2014 study surveying 380 medical students at an osteopathic medical school found that 15.2% had used prescription stimulants non-medically during medical school to aid in studying [15]. A 2021 literature review found that, across fourteen studies, 970 of 11,029 medical students had used prescription stimulants without a prescription [14].

Most prescription stimulant misusers obtain the medication via diversion, which involves obtaining medication from a peer with a prescription [6]. Malingering, or exaggerating one’s symptoms to obtain a prescription from a healthcare provider, is another method in which individuals seek medication. A 2005 nationally administered survey of over 4,000 adults reported that among those with a history of nonmedical use within the past year, approximately 20% obtained the medication through misrepresenting symptoms to a doctor [7]. More recently, a 2020 study surveying college students with stimulant prescriptions estimated that 55% had malingered or exaggerated their symptoms [16].

The vast majority of stimulants are prescribed for the treatment of ADHD [17]. Symptoms of ADHD include distractibility, restlessness, difficulty sustaining attention for tasks, forgetfulness, talking excessively, interrupting others while speaking, and losing or misplacing objects [18]. The diagnosis of ADHD requires symptoms to be present before the age of 12, persist for at least 6 months, have a significant impact on daily functioning, and occur in more than one setting [18]. Although ADHD is considered a childhood psychiatric disorder, many patients are not diagnosed until adulthood. It is recommended for providers to use supplemental tools to evaluate for ADHD in adults, in addition to the diagnostic criteria provided by the Diagnostic and Statistical Manual of Mental Disorders (DSM-5) [19]. For instance, the Continuous Performance Task (CPT) provides objective performance data measuring inattention and impulsivity [19]. More feasible methods of obtaining less subjective data include inquiry of a patient’s childhood and academic history.

A national survey found that the prevalence of diagnosed ADHD among U.S. children and adolescents increased from 6.1% in 1997 to 10.2% in 2016 [20,21]. Although improved detection is likely a contributing factor, there has been significant public concern about misdiagnosis. The diagnosis of ADHD relies on accurate and unbiased patient reporting, as well as on the application of DSM-5 criteria, which may be interpreted differently by providers. Many core symptoms of ADHD are nonspecific, increasing the risk of misdiagnosis when they are attributed to ADHD rather than to another underlying medical condition [22]. For instance, depression and burnout are highly prevalent among medical students and often present with impaired concentration and poor attention [23]. Misdiagnosis may result from incomplete clinical evaluation, misattribution of symptoms, or intentional or unintentional exaggeration of symptom severity by the patient. A 2017 study found there to be increased rates of self-reported disabilities, such as ADHD, among U.S. medical students compared to previous years [24]. However, whether this is primarily a consequence of improved detection or misdiagnosis remains questionable.

Prescription stimulant misuse is an increasingly prevalent public health issue. While medical students are a population at particularly high risk, limited data exist surrounding this group. Existing studies focus primarily on nonspecific variables, such as motivations for use or methods of obtaining medication. To date, little research has evaluated factors unique to medical students, a perspective that may ultimately lead to more targeted and effective preventive strategies.

Considering this, the primary objective of this cross-sectional survey was to examine factors associated with prescription stimulant misuse among medical students, encompassing individual attitudes and behaviors as well as prescribing practices of providers. Attitudes and behaviors assessed included perceived safety of prescription stimulants, academic pressure to engage in use to compete with peers, knowledge of medication adverse effects and legal consequences of diversion, and perceived normalcy of misuse among peers. Prescribing practices were evaluated through components including appropriate ADHD assessment and diagnosis, timing of initial prescription, and the provision of medication counseling. 

Secondary objectives included identifying variables associated with potential future misuse of prescription stimulants among non-users and comparing side effects, usage patterns, motivations for use, malingering, and diversion behaviors between students with and without a prescription.

## Materials and methods

Study sample

MD and MD/PhD students at least 18 years of age and enrolled at the Penn College of Medicine at the time of data collection were eligible for inclusion in the study. Approximately 600 students were eligible for inclusion. Students who did not engage with or complete the survey were excluded from the study. An electronic link to the 72-item online survey, administered via REDCap, was distributed to potential participants (approximately 600 medical students) via university email (census sampling). Eligible participants were identified using the medical school’s email distribution list for medical student classes enrolled at the time of survey distribution. Participants were enrolled in the study from March 5th, 2023, to April 2nd, 2023. The study was approved and deemed exempt by the Penn State Institutional Review Board (IRB) on 2/9/2023.

Data collection

A recruitment email was sent to eligible individuals at the beginning of data collection. It provided the purpose of the study, the time period for survey collection, a link to the REDCap survey, and details regarding potential financial compensation. At the conclusion of the study, one hundred randomly selected participants were awarded a ten-dollar Amazon gift card. Respondents indicated a preference for potential financial compensation at the start of the survey, and provided an email address for purposes of gift card transfer, if so. Email addresses were used to facilitate financial compensation and were not associated with survey responses. No other personal identifiable information was collected. A reminder email was sent seven days prior to the closure of the survey.

Variables

The survey instrument was developed by the investigators based on a review of existing literature on prescription stimulant misuse and prescribing practices. Content validity was established through expert review by faculty with internal medicine and addiction medicine training. The survey was pilot-tested among a small group of medical students to assess clarity and comprehension prior to distribution. Formal psychometric validation was not conducted as part of this study.

The survey collected self-reported demographic variables, including age, gender, ethnicity, and year of medical education at the time of survey completion. The latter was divided into five groups, including first-, second-, third-, and fourth-year MD students, and MD/PhD students of all classes. Participants indicated up to three residency specialties of interest and reported a history of prescription stimulant use as a medical student. They were subsequently divided into one of three groups: prescription stimulant users with a prescription, prescription stimulant users without a prescription, and non-users.

Using Likert-style questions, participants reported their understanding of the long term health risks of prescription stimulant use, understanding of legal consequences of medication diversion, likelihood of a change in understanding of medication health risks or behavior of engaging in use if more education on prescription stimulants were provided by medical school, frequency in which one felt pressure to use prescription stimulants to compete academically with other student users, frequency of witnessing peers engage in prescription stimulant misuse, and agreeability that prescription stimulants are safe. Additional questions posed in the survey were dependent on the participant group and are displayed in Table 1.

**Table 1:** Survey questions for participant groups ADHD: Attention Deficit Hyperactivity Disorder

Prescription Stimulant Users with Prescription	Prescription Stimulant Users without Prescription	Non-Users
Perceived reliance on medication for academic performance	Perceived reliance on medication for academic performance	History of prescription stimulant use before medical school
History of malingering	History of malingering	History of being offered medication
Medical reason for prescription	Agreement that one would obtain medication if feasible	Agreement that one would obtain medication if feasible
History of formal evaluation for ADHD	Agreement that one would engage in malingering to receive prescription	Agreement that one would engage in malingering to receive prescription
Motivations for use	Motivations for use	
Experienced side effects	Experienced side effects	
Dosage + frequency of administration	Dosage + frequency of administration	
Route of administration	Route of administration	
Age at initial prescription	Methods of obtaining medication	
History of medication diversion	Age at first time of prescription stimulant misuse	
Motivations and frequency of diversion		
History of prescription stimulant misuse		
Specialty of provider who issued initial prescription, if given for ADHD		
History of receiving medication counseling at initial prescription		
History of receiving legal counseling at initial prescription		
Perceived likelihood of behavioral change with medication counseling		
Perceived likelihood of behavioral change with legal counseling		
History of being asked by peer for medication		

Statistical analyses

All variables were summarized prior to analysis to assess their distributions. Group comparisons for categorical variables were made using Chi-square tests. Exact chi-square tests were used as necessary when the assumptions for an asymptotic test were not met. Group comparisons for Likert scale variables and continuous variables that were not normal were made using Wilcoxon Rank Sum tests (2 groups) or Kruskal-Wallis tests (more than 2 groups). All comparisons used a significance level of 0.05. P-values from the pairwise group comparisons of the Kruskal-Wallis tests were adjusted for multiple comparisons using the Dwass-Steel-Critchlow-Fligner method. Spearman correlation was used to look for correlations between continuous variables using a null hypothesis of ≤ 0.5 or ≥ -0.5 and an alternative hypothesis of > 0.5 or < -0.5 for significance. All analyses were performed using SAS software version 9.4 (SAS Institute, Cary, NC) and were conducted by a biostatistician.

## Results

Participant demographics

**Figure 1:**
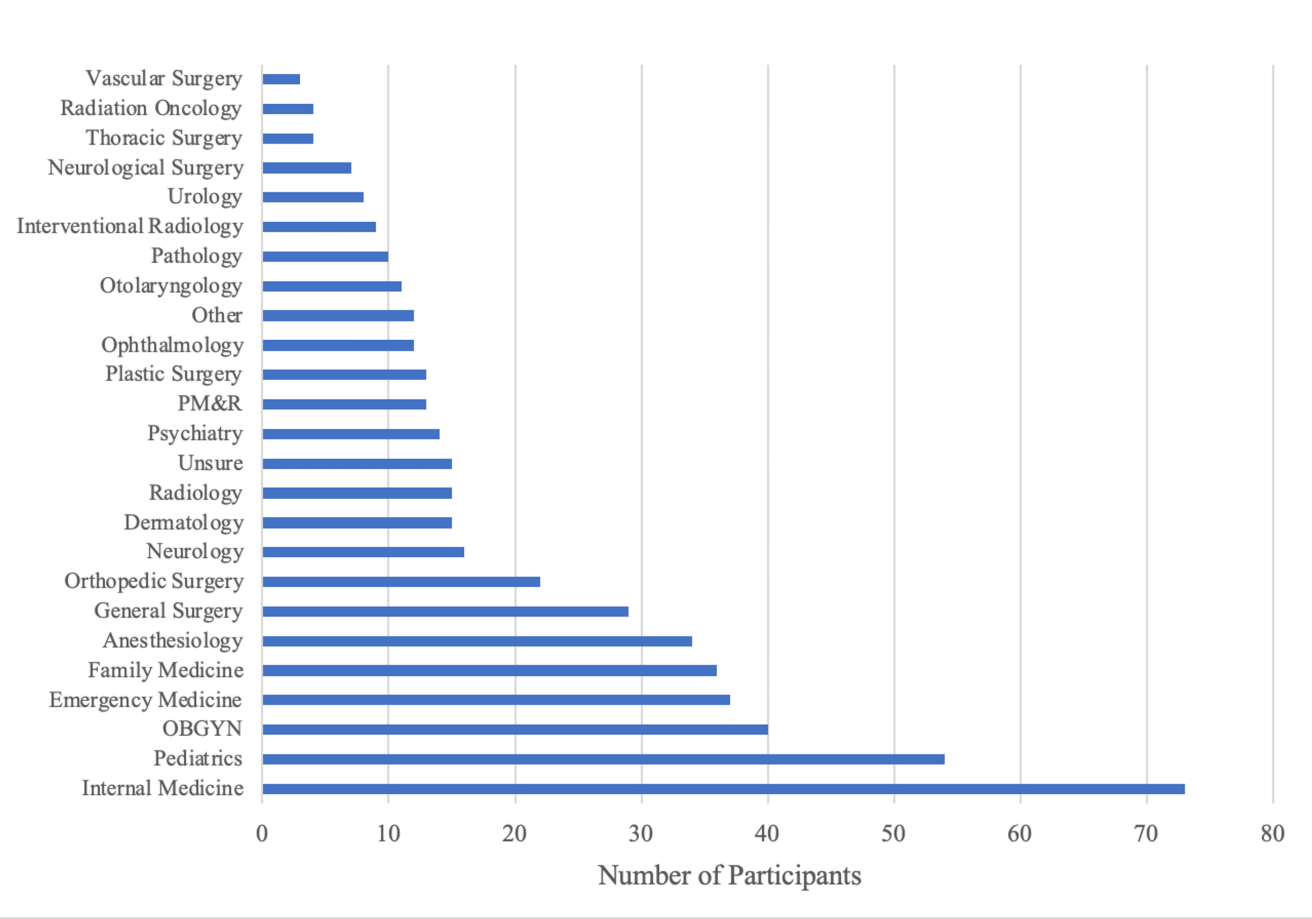
Residency interests of participants Participants were asked to indicate up to three specialties they were interested in. 73 participants (25.2%) reported an interest in internal medicine, 54 (18.6%) pediatrics, 40 (13.8%) obstetrics & gynecology, 37 (12.8%) emergency medicine, 36 (12.4%) family medicine, 34 (11.7%) anesthesiology, 29 (10.0%) general surgery, 22 (7.6%) orthopedic surgery, 16 (5.5%) neurology, 15 (5.2%) dermatology, 15 (5.2%) diagnostic radiology, 14 (4.8%) psychiatry, 13 (4.5%) physical medicine & rehabilitation, 13 (4.5%) plastic surgery, 12 (4.1%) ophthalmology, 11 (3.8%) otolaryngology, 10 (3.5%) pathology, 9 (3.1%) interventional radiology, 8 (2.8%) urology, 7 (2.4%) neurological surgery, 4 (1.4%) thoracic surgery, 4 (1.4%) radiation oncology, 3 (1.0%) vascular surgery, 12 (4.1%) a specialty not listed, and 15 (5.2%) being unsure.

A total of 290 students engaged with and completed the survey, with a 48.3% response rate. Self-reported demographics of participants are displayed in Table 2. Reported interest in medical specialties is shown in Figure 1. Since starting medical school, 34 respondents (11.7%) reported having used a prescription stimulant, and 256 respondents (88.3%) denied use. Of the 34 student users, 29 (85.3%) had a prescription and 5 (14.7%) did not.

**Table 2:** Participant demographics Total participants (N=290)

Variables	
Age, mean (SD)	25.58 (2.52)
Sex, N (%)	
Female	202 (69.66%)
Male	83 (28.62%)
Non-binary	2 (0.69%)
Prefer not to disclose	3 (1.03%)
Ethnicity, N (%)	
White	192 (66.21%)
Black/African American	4 (1.38%)
Asian	65 (22.21%)
Hispanic or Latino	4 (1.38%)
Multiracial/Biracial	13 (4.48%)
Other	3 (1.03%)
Prefer not to disclose	9 (3.10%)
Current year of medical school, N (%)	
MS1	81 (27.93%)
MS2	55 (18.97%)
MS3	67 (23.10%)
MS4	71 (24.48%)
MD/PhD (all classes)	16 (5.52%)

Stimulant users with a prescription

For users with a prescription, the mean age at the time of initial prescription was 20.72 (SD = 5.24) years. Of these participants, 23 (79.31%) received a stimulant prescription for ADHD (evaluated and diagnosed by a licensed psychologist or psychiatrist), 2 (6.9%) for ADHD (evaluated and diagnosed by another specialty provider), 3 (10.34%) for ADHD-like symptoms without an established diagnosis, and 1 (3.45%) for narcolepsy. The two unspecified healthcare providers were in family medicine and pediatric specialties. Table 3 displays additional characteristics assessed, including a history of receiving medication and legal counseling by a provider, as well as a history of malingering, medication diversion, and misuse.

**Table 3:** Prescription stimulant users ADHD: Attention Deficit Hyperactivity Disorder

Characteristic	Frequency (%)
Reason for Prescription	
ADHD	25 (86.2%)
Evaluating Provider	
Psychiatrist or Psychologist	23 (92.0%)
Family Medicine Physician	1 (4.0%)
Pediatrician	1 (4.0%)
Symptoms of ADHD without diagnosis	3 (10.3%)
Narcolepsy	1 (3.5%)
Obesity	0 (0.0%)
Malingering for Diagnosis	
Yes	2 (6.9%)
No	27 (93.1%)
Received First Prescription	
Prior to Medical School	17 (58.6%)
During Medical School	12 (41.4%)
Received medication counseling	
Yes	16 (55.2%)
No	13 (44.8%)
Received legal counseling	
Yes	15 (51.7%)
No	14 (48.3%)
History of peer asking for medication	
Yes	4 (13.8%)
No	25 (86.2%)
History of diversion	
Yes	2 (6.9%)
No	27 (93.1%)
History of medication misuse	
Yes	3 (10.3%)
No	26 (89.7%)

**Figure 2:**
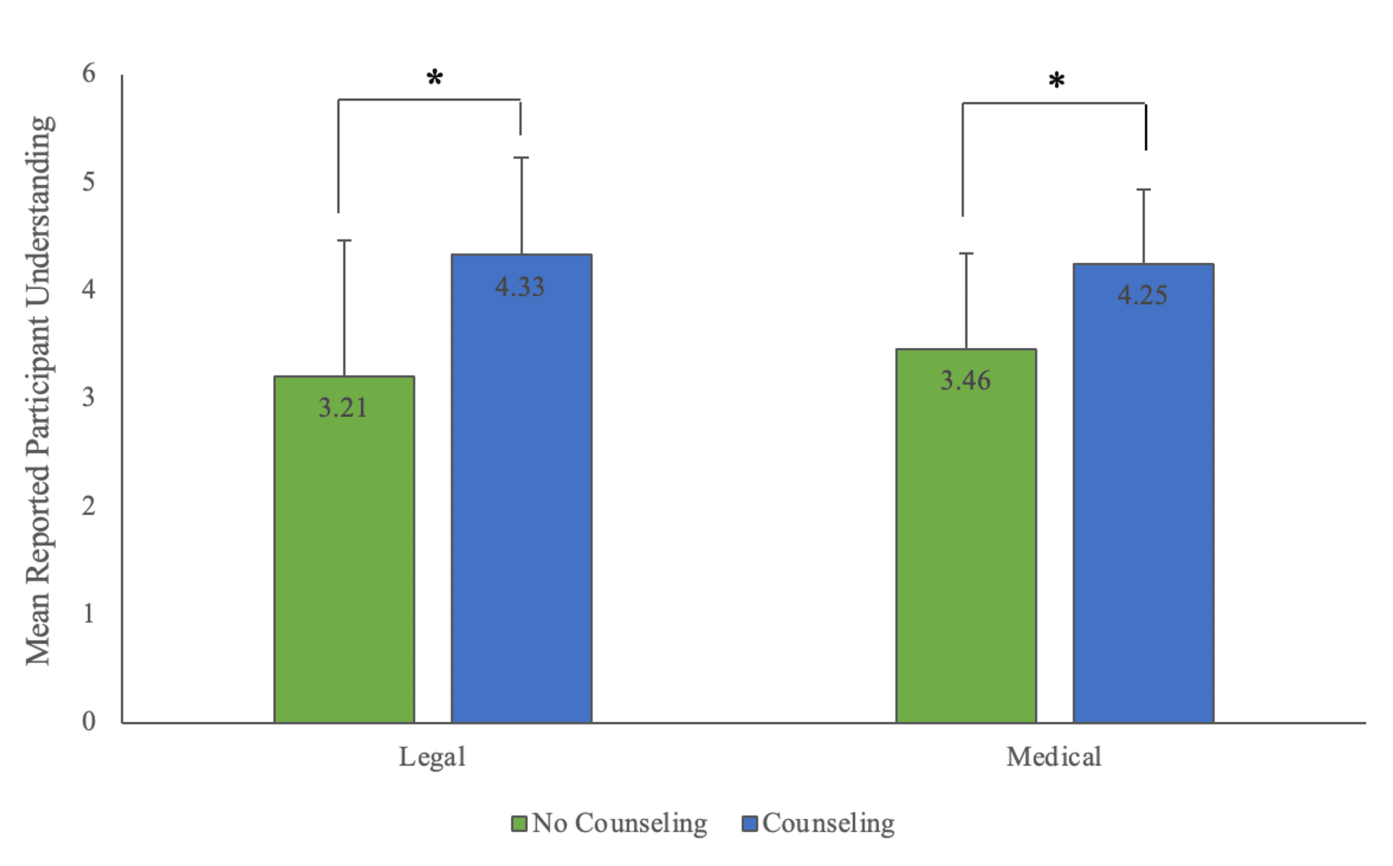
Prescription stimulant counseling and knowledge of medication health risks and consequences of diversion. Prescription stimulant users with a prescription who received legal counseling at time of first prescription had a significantly higher mean reported understanding of legal consequences of medication diversion (M = 4.33, SD = 0.90, 95% CI [3.84, 4.83]) compared to those who did not receive counseling (M = 3.21, SD = 1.25, 95% CI [2.49, 3.94]) (p = 0.012). Those who received medication counseling had a significantly higher mean reported understanding of medication adverse effects (M = 4.25, SD = 0.68, 95% CI [3.89, 4.61]) compared to those who did not (M = 3.46, SD = 0.88, 95% CI [2.93, 3.99]) (p = 0.017). Mean reported understanding is displayed at end of bars, with green bars representing those who did not receive counseling and blue bars those who did. Error bars represent standard deviation from the mean. Statistically significant differences are marked with an asterisk.

Medication counseling was defined as counseling on the adverse health effects of medication use. Legal counseling was defined as counseling on medication diversion and the legal implications of doing so. Of the 12 students who received their first prescription during medical school, 6 (50.0%) received medication counseling, and 5 (41.7%) received legal counseling. There was no statistically significant association between the timing of initial prescription (before vs. during medical school) and history of receiving medication counseling (χ²(1, N = 29) = 0.221, p = 0.64) or legal counseling (χ²(1, N = 29) = 0.829, p = 0.36) by prescribing provider. Those who received legal counseling had a significantly higher understanding of the legal consequences of diversion (M = 4.33, 95% CI [3.84, 4.83]) than those who did not (M = 3.21, 95% CI [2.49, 3.94]), p = 0.012. Likewise, students who received medication counseling had a significantly higher understanding of prescription stimulant health risks (M = 4.25, 95% CI [3.89, 4.61]) compared to students who did not receive medication counseling (M = 3.46, 95% CI [2.93, 3.99]), p = 0.017 (Figure 2).

Malingering

Of the two students who malingered, one received their first prescription after being diagnosed with ADHD by a trained professional, while the second received theirs before medical school for ADHD symptoms alone, without an evaluation or formal diagnosis. Students who engaged in malingering reported a non-significantly higher average experienced pressure to use prescription stimulants (M = 2.00, 95% CI [-10.71, 14.71]) compared to students who did not malinger (M = 1.96, 95% CI [1.40, 2.53), p = 0.885. A history of malingering was not associated with the number of medication side effects (p = 0.927), reliance on medication for academic performance (p= 0.930), or motivations for use (p = 0.325).

Diversion

The two respondents with a history of diversion had done so a total of 1-5 times. Both reported that they provided medication to a family member or friend without any drive for financial gain or fear of social rejection. Of the 29 users, both individuals with a history of medication diversion did not receive legal counseling from their prescribing provider, whereas 15 of the 27 users without a history of diversion (55.6%) did receive legal counseling. An exact chi-square test indicated that this difference was not statistically significant, χ²(1, N = 29) = 2.30, p = 0.215. Both participants with a history of diversion reported that legal counseling would likely have altered their diversion behavior. An exact chi-square test found no significant association between a history of medication diversion and malingering to obtain prescriptions, χ²(1, N = 29) = 6.22, p = 0.137.

Misuse

Of the three participants with a prescription who misused medication (i.e., took medication at a higher dose or frequency than prescribed), two had prescriptions for a diagnosis of ADHD and one for symptoms of ADHD without an established diagnosis. None of these three participants received medication counseling from their provider, whereas 16 of the 26 participants without a history of misuse (61.5%) did receive counseling. An exact chi-square test found that this difference was not statistically significant, χ²(1, N = 29) = 4.12, p = 0.08. Students with a history of misuse reported experiencing greater pressure to use prescription stimulants to compete academically with peers who also use medication (M = 3.00, 95% CI [-1.97, 7.97]) compared to students without a history of misuse (M = 1.85, 95% CI [1.31, 2.38]), though the difference was not statistically significant (p = 0.244). History of misuse was not significantly associated with malingering, χ²(1, N = 29) = 3.64, p = 0.198, nor with reason for prescription, χ²(3, N = 29) = 2.12, p = 0.52.

Stimulant users with vs. without prescription

Three of the five prescription stimulant users (60.0%) without a prescription used medication prior to medical school. All denied a history of malingering as an attempt to receive a prescription. Four of the five individuals (80.0%) obtained medication from a peer with a prescription, while one (20.0%) obtained it from a family member. Table 4 displays behaviors of medication use, motivations for use, experienced side effects, and reported reliance on medication for academic performance for stimulant users with and without a prescription. Statistical tests were conducted on all variables displayed, excluding the frequency of medication use and route of administration. Users with a prescription reported a significantly higher reliance on medication (M = 2.90, 95% CI [2.32, 3.47]) compared to users without a prescription (M = 1.40, 95% CI [0.29, 2.51]), p = 0.04. In addition to selecting all applicable motivations for medication use, participants were asked to indicate their primary motivation. Of users with a prescription, 13 participants (44.8%) indicated their primary motivation to be enhanced concentration while studying, 14 (48.3%) a diagnosed medical condition, 1 (3.4%) improved academic performance on exams, and 1 (3.4%) a reason not specified. Of users without a prescription, 3 participants (60.0%) selected enhanced concentration while studying, 1 (20.0%) euphoria, and 1 (20.0%) a reason not specified. An exact chi-square test found that the primary motivation of use approached, but did not reach, statistical significance χ²(4, N = 34) = 10.58, p = 0.057. There was no statistically significant difference in the mean number of reported side effects between users with a prescription (M = 2.55, 95% CI [1.75, 3.35]) and those without (M = 1.80, 95% CI [0.76, 2.84]), p = 0.76.

**Table 4:** Behaviors of prescription stimulant use among users with vs. without a prescription. Table 4 displays route of medication administration, frequency of use, mean medication dose, motivations of use, experienced side effects, and reported reliance on medication for users with and without a prescription. Statistical tests were conducted on all variables displayed, excluding the frequency of medication use and route of administration. Users with a prescription reported a higher average medication dose (M = 21.48, 95% CI [14.21, 28.76]) compared to users without a prescription (M = 8.60, 95% CI [0.06, 17.14]), p = 0.095. Among respondents who selected “other” as a motivation, two users without a prescription reported “busy day” as their reason. Among the three users with a prescription who selected “other,” responses included improved organization and cleanliness, overcoming “brain fog,” perceived inability to keep up with peers without the medication, increased wakefulness, and concerns about feeling dependent on the medication. Users with a prescription reported a significantly higher reliance on medication (M = 2.90, 95% CI [2.32, 3.47]) compared to users without a prescription (M = 1.40, 95% CI [0.29, 2.51]), p = 0.037.

Characteristic	Frequency of Subjects (%)		P-value
	Prescription	No Prescription	
Route of Administration			
Oral	29 (100.0%)	5 (100.0%)	
Other	0 (0.0%)	0 (0.0%)	
Frequency of Use			
< 5x per year	1 (3.4%)	4 (80.0%)	
< 1-5x per month	2 (6.9%)	1 (20.0%)	
1-2x daily	24 (82.8%)	0 (0.0%)	
> 2x daily	1 (3.4%)	0 (0.0%)	
N/A	1 (3.4%)	--	
Mean Dosage	21.48 (SD=19.1)	8.60 (SD=6.9)	0.095
Motivations of Use			
Enhanced concentration while studying	21 (72.4%)	4 (80.0%)	1
Improved academic performance on exams	8 (27.6%)	1 (20.0%)	1
Increased alertness on clinical rotation	8 (27.6%)	0 (0.0%)	0.304
Prolonged wakefulness for studying	5 (17.2%)	1 (20.0%)	1
Euphoria	1 (3.4%)	1 (20.0%)	0.277
Weight loss	1 (3.4%)	0 (0.0%)	1
Diagnosed medical condition	24 (82.8%)	--	--
Other	3 (10.3%)	2 (40.0%)	0.149
Side Effects			
Heart palpitations	11 (37.9%)	0 (0.0%)	0.145
Anxiety	9 (31.0%)	1 (20.0%)	1
Insomnia	8 (27.6%)	0 (0.0%)	0.304
Loss of appetite	13 (44.8%)	3 (60.0%)	0.648
Weight loss	2 (6.9%)	0 (0.0%)	1
Diarrhea/GI upset	4 (13.8%)	1 (20.0%)	1
Irritability	4 (13.8%)	0 (0.0%)	0.6
Depressed mood	2 (6.9%)	0 (0.0%)	1
Headache	7 (24.1%)	0 (0.0%)	0.33
Dizziness	2 (6.9%)	0 (0.0%)	1
Euphoria	12 (41.4%)	4 (80.0%)	0.168
Drug Reliance	2.90 (SD=1.5)	1.40 (SD=0.9)	0.04

Non-users vs. stimulant users without a prescription

Of the 256 individuals who denied current prescription stimulant use, 13 (5.1%) reported use prior to medical school. Fifty-one respondents (19.9%) had been offered a prescription stimulant at least once in their lifetime. Among non-users, there was a statistically significant correlation between pressure to use medication and agreeability one would obtain medication if it were available (+0.387, p < 0.0001). A positive correlation was seen among stimulant users without a prescription; however, not significant (+0.250, p = 0.580). Comparing pressure to use medication and reported likelihood of malingering, a significant positive correlation was seen among non-users (+0.422, p < 0.0001) and a stronger, yet non-significant correlation, among users without a prescription (+0.589, p = 0.170).

Additional outcomes

Year of medical school education was significantly associated with behaviors of prescription stimulant use, as described in Table 5 (χ²(8, N = 290) = 17.65, p = 0.027). Participants who experienced greater pressure to use prescription stimulants were significantly more likely to indicate an interest in several residencies. These included psychiatry (p = 0.036), obstetrics & gynecology (p = 0.025), interventional radiology (p = 0.044), urology (p < 0.001), and physical medicine & rehabilitation (PM&R) (p = 0.050) residencies. Table 6 displays additional comparisons made among groups. Compared to non-users, users with a prescription reported significantly higher pressure to use medication (χ²(1, N = 285) = 3.73, p = 0.027), perceived safety of medication (χ²(1, N = 285) = 5.97, p < 0.001), projected change in knowledge and behavior of medication use if more education were provided by medical institution (χ²(1, N = 285) = 3.71, p = 0.024), understanding of legal consequences of diversion (χ²(1, N = 285) = 5.75, p < 0.001), and understanding of medical risks of use (χ²(2, N = 285) = 3.63, p = 0.028). In addition, users without a prescription reported a significantly higher likelihood of malingering (M = 2.40, 95% CI [0.98, 3.82]) compared to non-users (M = 1.46, 95% CI [1.37, 1.56]), p = 0.014. When comparing the frequency of witnessing peers use stimulant medications without a prescription and the pressure to use medication, a significant positive correlation was found among non-users (+0.336, p < 0.0001). Non-significant correlations were found among users with a prescription (+0.305, p = 0.0595) and users without (-0.354, p = 0.421).

**Table 5:** Medical school class & prescription stimulant use.

Class	Frequent of Subjects (%)		None
	Prescription	No Prescription	
MS1	9 (11.1%)	2 (2.5%)	70 (86.4%)
MS2	7 (12.7%)	0 (0.0%)	48 (87.3%)
MS3	6 (9.0%)	0 (0.0%)	61 (90.0%)
MS4	2 (2.8%)	3 (4.2%)	66 (93.0%)
MD/PhD	5 (31.3%)	0 (0.0%)	11 (68.8%)

**Table 6:** Comparisons among groups Comparisons among users with a prescription, users without a prescription, and non-users were conducted using the Kruskal–Wallis test. Group comparisons were adjusted using the Dwass–Steel–Critchlow–Fligner method. Outcomes included perceived academic pressure to use prescription stimulants, beliefs regarding medication safety, reported understanding of medication adverse effects and legal implications of diversion, anticipated changes in knowledge of medical and legal consequences, and in medication-related behaviors (e.g., frequency of use, diversion), with additional medical school education, and reported likelihood of malingering if doing so would guarantee obtaining a prescription from a healthcare provider.

Outcome	Non-users	Prescription	No Prescription	Non-users vs. Prescription	Prescription vs. No Prescription	Non-users vs. No Prescription
	N=256	N=29	N=5			
	Mean ± SD	Mean ± SD	Mean ± SD	P-value	P-value	P-value
Pressure to Use Medication Higher=greater pressure	1.33 ± 0.69	1.97 ± 1.40	2.00 ± 1.00	0.024	0.891	0.09
Prescription Stimulants Safe Higher=more agreement	2.95 ± 0.88	3.66 ± 0.55	3.40 ± 0.55	<0.001	0.608	0.494
Understanding of Adverse Effects Higher=more understanding	3.32 ± 1.13	3.90 ± 0.86	4.00 ± 0.71	0.028	0.986	0.391
Understanding of Legal Consequences of Diversion Higher=more understanding	2.74 ± 1.27	3.79 ± 1.21	3.80 ± 1.30	<0.001	0.999	0.171
Predicted Increase in Knowledge/Behavior if Provided More Education Higher=greater increase	3.16 ± 0.96	2.59 ± 1.15	3.00 ± 1.00	0.024	0.73	0.922
Likelihood of Malingering Higher=more likely	1.46 ± 0.81	N/A	2.40 ± 1.14	N/A	N/A	0.014

## Discussion

One of the primary objectives of this study was to characterize stimulant prescribing practices, as inadequate clinical evaluation, misdiagnosis, and failure to detect signs of malingering may contribute to prescription stimulant misuse. Our results indicate that inappropriate prescribing practices do exist in our study population. Four students obtained a prescription either through malingering or because of an insufficient ADHD evaluation by their provider. The latter group was too small to adequately assess if certain specialties were more likely than others to perform insufficient assessments. This would be an interesting topic for larger studies to explore; if certain specialty providers are associated with these behaviors, they may benefit from the use of supplemental assessment tools. It is important to recognize, however, that our survey did not provide an operational definition of what constitutes an “adequate assessment,” ultimately leaving room for subjectivity in participants’ responses. The most accurate method to assess the adequacy of ADHD evaluations is observation of clinical evaluations in real time. Unfortunately, the feasibility of this is limited. Future studies could better assess this variable by including operational definitions if survey-based, or by employing alternate methodologies that gather data from the providers themselves [25]. A benefit of the latter approach is having the opportunity to explore unconscious biases providers may have towards patients in medical training. Although assessment of clinical evaluations is challenging and potentially better suited for prospective data collection, it would be worthwhile for future studies to pursue. Our data reveal the presence of inappropriate prescribing, which warrants more attention. Collecting more standardized data from a larger study population would offer more objective and generalizable insight into the impact of inappropriate prescribing practices on prescription stimulant misuse.

Unsurprisingly, receiving provider counseling was associated with a significantly greater understanding of medication health risks and the consequences of diversion. However, we found no significant relationship between these measures of understanding and the behaviors of misuse or diversion. When analyzing the history of counseling with misuse and diversion, there were several notable findings. Compared to individuals without a history of behavior, none of the three prescription users with a history of misuse received medication counseling (p = 0.08) and neither of the two individuals with a history of diversion received legal counseling (p = 0.22). This should be interpreted with caution, however, because the lack of significance may reflect insufficient sample size rather than a true absence of association. Nonetheless, a relationship is plausible and has been supported in the literature. A 2020 study of primary care prevention training for stimulant misuse reported that improved provider-patient education significantly reduced patients’ intent to divert and non-significantly reduced the frequency of diversion [26]. Although it was the intention, rather than the behavior itself, that was significantly reduced, the results still underscore the value of provider-patient education. In this study, both users with a history of diversion reported that they likely would have changed this past behavior if they had received legal counseling at the time of initial prescription. This provides at least some qualitative data suggesting that provider counseling influences behavior.

Although this study lacked statistical power to detect a statistically significant association, we believe that understanding the medical risks of prescription stimulants and the legal consequences of diversion does influence individuals’ willingness to engage in misuse and diversion, respectively. As such, it is concerning that just over half of prescription users received medication and legal counseling at the time of initial prescription. It was hypothesized that providers may be less inclined to counsel patients in medical school because they assume, consciously or subconsciously, that it is unnecessary. We found no significant association between the timing of the first prescription (before vs. during medical school) and history of counseling to suggest that this may be the case. It is more likely that broader factors account for this observed shortcoming. The literature suggests that insufficient provider counseling is an issue not unique to stimulant medication or medical students as a patient population. Many studies have reported that healthcare providers do not educate patients on the side effects of prescription medications as frequently as recommended [27]. Unfortunately, there is limited research on reasons why. Regardless, efforts should still be made to increase medication counseling on a broad scale. If increased attention is drawn to this issue, specific factors may be identified to guide more targeted approaches.

Another primary objective of this study was to identify behaviors and attitudes of medical students that contribute to prescription stimulant misuse. Our findings regarding primary motivations for misuse mirror those of previous studies and showed no significant differences between user groups. The value medical students place on academic performance and the ability to study for prolonged periods of time will likely not change. Therefore, it is unlikely that interventions aimed at minimizing these motivations will have an impact on overall patterns of misuse. The perceived normalcy of misuse among peers, on the other hand, is a factor that may be more easily addressed. Among non-users, perceived normalcy of peer misuse of prescription stimulants was significantly associated with greater pressure to use medication for academic gain, which in turn was significantly associated with greater agreeability to partake in malingering or obtain medication if given the opportunity. This sheds light on the potential impact of interventions aimed at reducing the perceived normalcy of misuse among medical student peers, as these approaches may help prevent non-users from initiating misuse. It is important to acknowledge our limitations in making this conclusion, as our study measured the likelihood or intent of misuse, rather than the frequency of the behavior. No significant associations were found among users without a prescription; however, this may have been limited by sample size and does not imply that this group would not benefit from this approach. 

Interestingly, this study found that students pursuing certain residencies experienced a significantly higher pressure to use prescription stimulants compared to peers. These included urology, PM&R, OBGYN, psychiatry, and interventional radiology. At least 3 of these 5 specialties have experienced an increase in competitiveness in recent years, demonstrated by data from the National Resident Match Program [28]. Students may feel greater pressure to use prescription stimulants in order to build a competitive residency application. It takes significant time and cognitive effort to author multiple research publications and take on meaningful leadership positions. Students may believe that cognitive-enhancing medication is the only way to meet these demands. Although this study demonstrates an association rather than a direct relationship, it is the first to explore residency interest as a potential risk factor for student misuse. Our study also found the year of education to be associated with behaviors of medication use. Use of prescription stimulants without a prescription was localized to first- and fourth-year medical students. This may be because of the demanding nature of transitioning to medical school or applying to residency programs. Like other findings in our study, our sample size limits our ability to draw general conclusions. We encourage studies of larger size to investigate this further.

Approximately 1.7% of surveyed students engaged in prescription stimulant use in medical school without having a prescription. This is lower than what has been reported nationally, with studies citing ranges between 5.2% to 47.4% [11]. This study was limited to a single institution, so characteristics unique to this cohort may have contributed to differences from national data. The survey response rate was about 50%, so non-response bias may also have played a role in this discrepancy. It is also possible that more students would have been included in this group, as a few were prescribed medication without an established diagnosis or because of malingering. This is all speculation, of course.

In 2024, the Centers for Disease Control and Prevention (CDC) estimated that approximately 6% of American adults have a diagnosis of ADHD [29]. After excluding the case of malingering, there were 24 students with an established diagnosis of ADHD. This was approximately 8% of the study cohort. This is slightly higher than national trends, although most studies are of the general adult population. Very little to no data exist on the prevalence of ADHD among medical students. Interestingly, 10 of these students were not diagnosed until medical school. Of these 10 students, four were diagnosed at the age they started medical school, and an additional four were diagnosed one year later. This timing suggests that the increased academic demands of medical school may unmask ADHD symptoms that were previously managed or compensated for. Still, considering that just under half of ADHD cases were diagnosed during medical school, this raises the question of whether medical school was truly the tipping point for all these individuals or if some were misdiagnosed.

Diagnosing ADHD in medical students poses many challenges. Since they are adults, the provider must establish the presence of symptoms in childhood. This should, ideally, involve objective data from parents or previous teachers. Since medical students exhibit high rates of depression and burnout, the clinician must also rule out other potential causes of their symptoms. The findings of a 2019 prospective study highlight the challenges of evaluating for ADHD in adult patients and how it may contribute to rates of misdiagnosis. The study followed 239 children without ADHD through adolescence and into adulthood to characterize cases of late-onset ADHD [30]. Of the 40 participants who met symptom and impairment criteria for ADHD during adulthood, only 2 cases were true diagnoses. Multi-informant observations, behaviors of substance abuse, co-existing psychiatric disorders, and evidence of impairment were utilized to identify false positive cases. [30]. This raises the concern that clinicians with limited ability to assess patient comorbidities and patterns of substance use are likely to misdiagnosis some adults with ADHD [30]. Since our study did not gather data on the circumstances of clinical assessments, we cannot make assumptions on how the presence or absence of these data points contributed to the rate of misdiagnosis. This is also well outside the scope of this study. Since recent data have reported rising cases of ADHD among medical students, it would be worthwhile for studies to investigate if misdiagnoses contribute to this trend.

## Conclusions

This study aimed to investigate prescribing practices, attitudes, and behaviors that may contribute to prescription stimulant misuse among medical students. Our findings reveal the presence of inappropriate prescribing, either from insufficient ADHD evaluations or the inability to identify signs of malingering. Although the circumstances of assessments were self-reported, these results highlight the need for improved clinician evaluations. A history of receiving counseling was associated with greater knowledge of medication health risks and consequences of diversion, which likely influence behaviors of misuse or diversion, although not seen in this study. Given that a low proportion of prescription users received counseling, efforts should be taken to increase provider medication counseling. Minimizing the perceived normalcy of prescription stimulant misuse among peers may be effective in reducing the number of students who initiate misuse to compete academically. Residency specialty interest and year of medical school education are potentially informative variables for identifying students at risk of misuse. Although the small sample size and single-institution design reduce generalizability, many of the variables examined have not been extensively studied in prior research. These findings may help guide more effective preventive strategies for prescription stimulant misuse and highlight promising avenues for future research.
